# Editorial: Fractals in the diagnosis and treatment of the retina and brain diseases

**DOI:** 10.3389/fnetp.2022.1054439

**Published:** 2022-10-18

**Authors:** Marina V. Zueva, Antonio Di Ieva, Svetlana D. Pyankova

**Affiliations:** ^1^ Department of Clinical Physiology of Vision, Helmholtz National Medical Research Center of Eye Diseases, Moscow, Russia; ^2^ Computational NeuroSurgery (CNS) Lab, Macquarie Medical School, Macquarie University, Sydney, NSW, Australia; ^3^ Faculty of Psychology, Lomonosov Moscow State University, Moscow, Russia

**Keywords:** fractal geometry and physiology, brain, retina, dendrites’ arborization, electroencephalogram—EEG, tumor surface regularity

Over the last decades, the non-linearity of natural objects has been shown to be suitable to the application of fractal analysis. By extending the principles of fractal geometry to the study of biology and to the human body, fractal physiology is receiving considerable research attention. The human body, including its most complex structure, i.e., the brain, is characterized by recursive systems and complex networks. Today it is well proven that the structural organization of the brain, the architecture of neural and vascular networks, and the dynamics of functional activity, amongst others, have fractal properties (Di Ieva, 2016). Advances in fractal physiology underlie technological innovation to address some of the pressing problems in medicine, eventually suggesting new diagnostic and therapeutic approaches. The spatial and temporal structure of incoming sensory information is likely the key factor on which human brain development and health depends.

Changes of objectively observed fractal characteristics are markers of age-related alterations, including the ones seen in normal and pathological aging and mental disorders as well. A change in the structural complexity, quantified by means of the fractal dimension, amongst other parameters, and of the dynamic patterns of activity of the brain and retina occur in diseases of various etiologies, including neurodegenerative disorders, brain tumors, cerebrovascular diseases, visual system’s disorders, and cognitive impairment during stress, chronic anxiety or depression. Moreover, the patterns of physiological and psychological responses to fractal stimulation therapy open the way for the formation of new therapeutic strategies.

This Issue is dedicated to computational fractal-based methodologies in medicine and applications of the achievements of fractal physiology in the diagnosis of the brain and retina disorders. An objective assessment of the fractal complexity of the structural and functional organization of the brain can provide new tools useful for differential diagnosis, novel prognostic tests to assess the brain and retina disorders and criteria to monitor the effectiveness of the therapeutic interventions.

Fractal dimension of the neuronal dendrites’ arborization was recently shown to be related to functional constraints - the need and ability of dendrites to compete to make connections to other neurons (Smith et al., 2021). Anterior thalamic nuclei lesions reduced spine density in hippocampal CA1 neurons, while an enriched environment increased spine density in both the experimental and control rats (Harland et al., 2014). The research by Rowland et al. raises the issue whether pathological states of neurons might affect this fractal branching optimization. Using confocal microscopy to obtain images of CA1 pyramidal neurons and constructing 3-dimensional models of the dendritic arbors, the authors found that control rats exposed to an enriched habitat and training in spatial memory showed higher dendrites branching complexity and connectivity compared to other conditions. Rats with or without anterior thalamic nuclei lesions both optimized the connectivity in respect to the material cost of competing connections. The authors used an improved technique on characterization of fractal dimension of dendritic pattern. The fractal dimension values to optimize these connections did not differ between rats’ groups with or without lesions, perhaps due to small morphological differences induced by these injuries. However, the results show that successful application of this method to characterize the dendritic pattern can become a promising diagnostic tool to help detect the neuronal pathological states that affect fractal optimization.

The tumor interface has an irregular geometry due to the complex dynamics of the tumor growth process, including the tumor cells proliferation of and the invasion of surrounding tissues. Fractal analysis is commonly used to characterize the complexity the tumor surface (Iftekharuddin et al., 2009; Di Ieva et al., 2015) and to monitor the effectiveness of therapy (Di Ieva et al., 2012). Sánchez and Martín-Landrove employed a method based on dynamic quantum clustering to perform contrast enhanced MRI of brain tumors and describe different morphological parameters including fractal dimension and lacunarity, growth dynamics exponents, regularity measures, and parameters derived from multifractal analysis. The tumor surface regularity, one-dimensional and bi-dimensional fluctuations of the tumor interface were analyzed by Detrended Fluctuation Analysis. Tumor interface fractal dimension, local roughness exponent and surface regularity were shown to be parameters that can discriminate between gliomas and meningiomas/schwannomas.

Currently, the attention of many researchers is attracted by the study of EEG in brain diseases (Racz et al., 2020), and the identification of neurophysiological markers of these pathological conditions. Dick and colleagues analyzed the degree of multifractality of EEG time series obtained in healthy subjects and patients with mental disorders (paranoid schizophrenia and depression) using the method of maximum modulus of the wavelet transform and multifractal detrended fluctuation analysis. The main differences between the multifractal properties of brain activity in healthy people and those with mental disorders were in the width of the multifractality spectrum and its location associated with the correlation or anticorrelation dynamics of the values ​​of successive time series. Schizophrenia was characterized by a greater degree of multifractality compared to depression. These data suggest that the degree of multifractality can be included in a diagnosis tool as the test for the differential diagnosis and further study of mental disorders.

More and more attention is now paid to the study of fractal functional connectivity in the brain. The effects of scale-dependent interactions make it difficult to analyze it. The assessment of the extent of the narrowband oscillatory components in combination with broadband fractal, which are presented in empirical data (e.g., EEG recording) is of great importance given that the fractal and oscillatory signal components may reflect different underlying processes (Buzsaki et al., 2012). For better assessment of fractal properties of brain oscillations and brain connectivity, it is necessary to separate the fractal component of the signal from the rest. Racz and coauthors introduced an extension of irregular-resampling auto-spectral analysis to the bivariate case - multiple-resampling cross-spectral analysis (MRCSA) to separate the fractal component of the cross-spectral spectrum of long-range coupled signals. The authors showed the applicability of MRCSA to EEG recordings by analyzing fractal connectivity at rest and under the condition of word generation.

The versatile applications of fractal analysis to the neurosciences, using the accumulated empirical data and existing theoretical models make it possible to identify promising future applications in the clinical setting and open new promising areas of research.
